# Production of biodegradable plastic by polyhydroxybutyrate (PHB) accumulating bacteria using low cost agricultural waste material

**DOI:** 10.1186/s13104-016-2321-y

**Published:** 2016-12-12

**Authors:** Anteneh Getachew, Fantahun Woldesenbet

**Affiliations:** 1 Department of Biotechnology, Wolkite University, Po. Box 07, Wolkite, Ethiopia; 2 Research Directorate, Arba Minch University, Po.Box 21, Arba Minch, Ethiopia

**Keywords:** Biodegradable, Bioplastic, FTIR, Polyhydroxybutyrates

## Abstract

**Background:**

Polyhydroxybutyrates (PHBs) are macromolecules synthesized by bacteria. They are inclusion bodies accumulated as reserve materials when the bacteria grow under different stress conditions. Because of their fast degradability under natural environmental conditions, PHBs are selected as alternatives for production of biodegradable plastics. The aim of this work was to isolate potential PHB producing bacteria, evaluate PHB production using agro-residues as carbon sources.

**Result:**

Among fifty bacterial strains isolated from different localities, ten PHB accumulating strains were selected and compared for their ability to accumulate PHB granules inside their cells. Isolate Arba Minch Waste Water (AWW) identified as *Bacillus* spp was found to be the best producer. The optimum pH, temperature, and incubation period for best PHB production by the isolate were 7, 37 °C, and 48 h respectively at 150 rpm. PHB production was best with glucose as carbon source and peptone as nitrogen source. The strain was able to accumulate 55.6, 51.6, 37.4 and 25% PHB when pretreated sugar cane bagasse, corn cob, teff straw (*Eragrostis tef*) and banana peel were used as carbon sources respectively. Fourier transform-infrared authentication results of the extracted and purified PHB identified its functional units as C–H, CH_2_, C=O and C–O groups. UV–Vis spectrophotometric analysis and biodegradability test confirmed the similarity of the extract with standard PHB and its suitability for bioplastic production.

**Conclusion:**

The isolated *Bacillus* sp can be used for feasible production of PHB using agro-residues especially sugarcane bagasse which can reduce the production cost in addition to reducing the disposal problem of these substrates. The yield of PHB can further be boosted by optimization of production parameters as substrates.

## Background

Plastic materials originated from petrochemicals cause serious environmental problems due to their non-degradable nature. Such synthetically produced polymers are generally inexpensive, but their persistence has a significant environmental impact [1]. With the imminent fossil fuel crisis, the alarming rate of petroleum prices and environmental impact associated with the products, the search for alternatives is essential in reducing mankind’s dependencies in non-renewable resources [2]. Biodegradable plastics offer the best solution to protect the environment from hazards caused by conventional petroleum based plastics as they are ‘eco-friendly’ in nature. There are many types of biodegradable plastics with different degrees of biodegradability. Among them polyhydroxybutyrate (PHBs) are the only 100% biodegradable ones.

PHBs are macromolecules synthesized by bacteria and are inclusion bodies accumulated as reserve material when the bacteria grow under different stress conditions [3]. They are polymers possessing properties similar to various synthetic thermoplastic like polypropylene. This makes them useful for extensive applications and future commercial mass production of biodegradable plastics that can replace plastic materials currently obtained from petroleum bases [4].

However, a major problem for extensive production and commercialization of PHBs is their high production cost as compared with plastics derived from petrochemicals. Recently, much effort has been committed to reduce the production cost of PHB by using strategies such as; developing efficient bacterial strains, optimizing fermentation and recovery processes [5, 6]. Most reports regarding the production of PHB suggested that, the major contributor to the overall PHB production cost was carbon substrate cost [7]. As such, the selection of efficient carbon substrate is a key aspect, which verifies the total cost of the final product. The alternative approach is to choose renewable, economically feasible and most readily available carbon substrates for both microbial growths and efficient PHB production [5, 7]. Therefore, the objective of the present study was to isolate PHB producing bacteria and study its PHB production from agricultural waste materials.

## Methods

### Isolation and preservation of bacterial strains

Samples were aseptically collected from different localities in and around Arba Minch city, serially diluted and inoculated into sterile nutrient agar plates. The plates were incubated at 37 °C for 24 h. Colonies with distinct features were picked and purified by repeated streaking on similar agar plates. The purified colonies were preserved on nutrient agar slants at 4 °C.

### Screening for PHA producing bacteria

The isolated colonies were screened for PHA production by Sudan black staining and ranked based on the magnitude of their staining according to Nandini et al. [8] and Burdon [9].

### Identification of PHA producing isolates

The PHA producing bacterial isolates were subjected to a set of morphological, physiological and biochemical tests and identified to the Genus level based on Bergey’s Manual of Determinative Bacteriology.

### Production of PHA by selected isolates

Mineral salts medium (MSM) [composition (g/L): Urea (1.0), Yeast extract (0.16), KH_2_PO_4_ (1.52), Na_2_HPO_4_ (4.0), MgSO_4_∙7H_2_O (0.52), CaCl_2_ (0.02), Glucose (40), and trace element solution 0.1 ml] was used for the production of PHA by the selected isolates. The trace element solution contained (g/L): ZnSO_4_∙7H_2_O (0.13), FeSO_4_∙7H_2_O (0.02), (NH_4_)_6_MO_7_O_24_. 4H_2_O (0.06) and H_3_BO_3_ (0.06). Both glucose and trace element solution were autoclaved separately, and reconstituted prior to inoculation.

The culture was prepared by sub culturing the isolates twice in nutrient broth. Then one ml of a 24 h old culture was inoculated into 100 mL production medium and incubated at 37 °C and 150 rpm for 48 h.

### Measurement of dry biomass

For dry biomass measurement the culture was centrifuged at 10,000 rpm for 15 min, and the pellet was dried in an oven at 55 °C to constant weight [10].

### Extraction and quantification of PHA

Ten mL of culture was centrifuged at 10,000 rpm for 15 min. The supernatant was discarded and the pellet was treated with 10 mL sodium hypochlorite and the mixture was incubated at 30 °C for 2 h. The mixture was centrifuged at 5000 rpm for 15 min and then washed with distilled water, acetone and methanol respectively The pellet was dissolved in 5 mL boiling chloroform and evaporated by pouring the solution on sterile glass tray kept at 4 °C and weighed. The relative PHB accumulation by the different isolates was compared to help in identification of the best producer.

### Effect of growth conditions on PHB production by selected isolate

The effect of initial pH (6.5, 7, 7.5 and 8), temperature (25, 30, 37 and 40 °C) and incubation period (up to 72 h in 12 h interval) on PHB production by the selected isolate was evaluated using MSM at 150 rpm. The initial pH of the medium was adjusted by 1 N hydrochloric acid or sodium hydroxide. The results were then compared by measuring the dry biomass and the weight of extracted PHA as described above.

### Effect of carbon sources on PHB production

The effect of glucose, fructose and sucrose on production of PHB by the selected isolate was evaluated by separately incorporating 4% (w/v) of the sugars in standard MSM at pH 7, 37 °C, 48 h and 150 rpm followed by dry biomass and extracted PHB weight measurements. Similar measurements were also made using pretreated Sugarcane bagasse, Corn cob, Teff (*Eragrostis teff*) straw and Banana peel with 4% (v/v) hydrolysates.

### Pretreatment of agricultural residues

Locally collected sugarcane bagasse, Corn cob, Teff (*Eragrostis teff*) straw and Banana peel were shredded into pieces, dried in oven at 60 °C for about 1 week and pulverized into fine particles. They were hydrolyzed by zinc chloride method as explained by Chen et al. [11]. The reducing sugar contents of their hydrolysates were estimated by Di-Nitrosalicylic acid (DNSA) method according to the method described by Miller [12].

### Effect of nitrogen sources on PHA production

The MSM was separately augmented with (1% w/v) nitrogen sources (peptone, urea, yeast extract, and ammonium sulphate) at pH 7 was inoculated with the isolate and incubated at 37 °C for 48 h and 150 rpm. The produced biomass and PHB were measured as above.

### FTIR spectrophotometer analysis of PHB

About 1 mg extracted sample of PHB was dissolved in 5 ml chloroform. After pellet was formed by adding KBr, spectra were recorded at 4000–400 cm^−1^ range by Spectrum 65 FT-IR (PerkinElmer) [13].

### UV–Vis spectrophotometer analysis of PHB

The extracted PHB was dissolved in chloroform and scanned in the range of 200–320 nm (UV/Vis spectrophotometer Rs-290) against chloroform blank and the spectrum was analyzed for a sharp peak at 240 nm [14].

### Preparation of a bioplastic film

Sample bioplastic film was prepared by dissolving 50 mg PHB extract in 10 ml chloroform according to Rawia et al. [15].

### PHB degradation study

Biodegradability of the polymer was studied by granule-agar suspension method of solidified medium according to Michael et al. [16]. PHB was incorporated in the test plate while a control plate was prepared without PHB. Both plates were inoculated with the same soil born bacteria and the plates were checked for clear zone formation.

### Statistical analysis

All the tests were conducted twice in triplicate and standard deviation was determined. The data was analyzed by one way ANOVA using Microsoft excel software 2007 to determine significance.

## Results

**Fig. 1:**
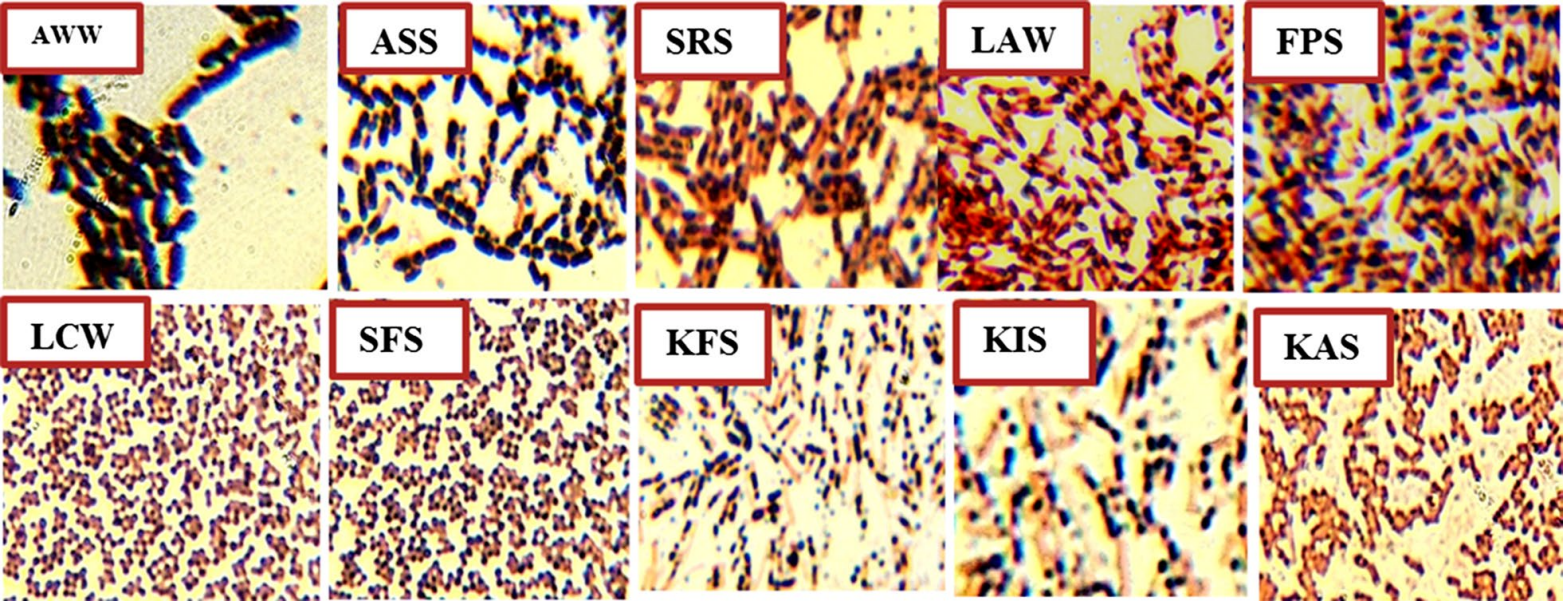
Photomicrograph of isolates showing the PHB granules produced in the form of dark granules in the bacterial cells

Among 50 strains of bacteria isolated from different places ten were found to be PHB producers with different relative PHB accumulation (Table 1 and Fig. 1). Most of the producers identified belonged to the genus *Bacillus* (Table 1).

**Table 1:** Morphological and biochemical characteristics used to classify the isolates

Isolate	Morphology		Biochemical properties					Probable genus
	Motility	Endospore	Gram	Catalase	Citrate	Indole	Starch hydrolysis	
AWW	+	+	+	*–*	+	*–*	*–*	*Bacillus*
ASS	+	+	+	*–*	+	*–*	*–*	*Bacillus*
SIS	+	*–*	+	+	+	+	+	*Bacillus*
LCW	+	+	+	+	*–*	+	+	*Micrococcus*
LAW	+	+	+	+	*–*	*–*	+	*Bacillus*
FPS	+	*–*	*–*	+	*–*	*–*	+	*Bacillus*
KFS	+	*–*	+	+	*–*	*–*	+	*Bacillus*
KAS	+	+	*–*	+	*–*	*–*	+	*Bacillus*
KIS	+	+	*–*	+	*–*	*–*	+	*Bacillus*
SFS	+	*–*	+	+	+	+	+	*Staphylococcus*

+ positive; – negative

**Fig. 2:**
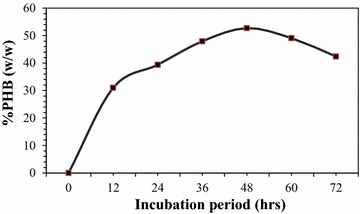
Time course of PHB production by isolate AWW at 37 °C and pH 7.0 using shake flasks at 150 rpm

Isolate AWW was taken for further studies as it produced higher PHB than the rest isolates (Table 2). As shown in Fig. 2 PHB production by isolate AWW increased up to 48 h incubation and declined afterwards.

**Table 2:** PHB accumulation by selected isolates at pH 7.0, 37 °C after 48 h and 150 rpm

Isolates	Growth OD600 nm	Dry biomass (g/l)	PHB (g/l)	Residual biomass (g/l)	PHB % (w/w)
AWW	1.159	12.0 ± 0.08	5.0 ± 0.44	7.0 ± 0.05	41.66
ASS	1.125	11.0 ± 0.00	3.9 ± 0.03	7.1 ± 0.13	35.45
KIS	0.991	9.4 ± 0.01	3.2 ± 0.07	6.2 ± 0.39	34.04
LCW	1.092	11.5 ± 0.11	3.9 ± 0.28	7.6 ± 0.66	33.91
LAW	1.021	9.0 ± 0.02	2.6 ± 0.07	6.4 ± 0.39	28.88
KIS	0.825	7.0 ± 0.38	2.0 ± 0.33	5.0 ± 0.12	28.57
FPS	0.981	8.9 ± 0.26	2.1 ± 0.43	6.8 ± 0.00	23.59
SRS	1.106	6.9 ± 0.09	1.4 ± 0.02	5.5 ± 0.57	20.28
SFS	0.646	5.5 ± 0.35	1.0 ± 0.02	4.5 ± 0.06	18.18
KAS	0.762	6.0 ± 0.00	1.0 ± 0.41	5.0 ± 0.01	16.66

Data represent two trials done in triplicates ± standard deviation

After the agro-residues were hydrolysed the reducing sugar contents of the hydrolysates were measured as 4715, 4465, 4215 and 3965 µg/ml for sugarcane bagasse; corn cob, teff straw and banana peel respectively.

The best pH and temperature for PHB production by the isolate were 7.0–7.5 and 37 °C. Among the carbon sources tested glucose was the best carbon source with 61% PHB production. Sugarcane bagasse hydrolysates resulted in 56% PHB production which is equivalent to fructose (54%). Corn cob and sucrose provided comparable production of PHB 52 and 49% respectively. Teff straw resulted in 39% production of PHB. The least PHB production was exhibited by banana peel with 27% PHB (Table 3).

**Table 3:** Effect of pH, temperature, carbon and nitrogen sources on production of polyhydroxybutyrate (PHB) by isolate AWW

Parameter/nutrient	Dry biomass (g/l)*	PHB (g/l)	% PHB (w/w)
Temperature (°C)			
37	12 ± 0.12^a^	6.8 ± 0.12^a^	56.66^a^
30	9.6 ± 0.21^b^	4.9 ± 0.21^b^	51.04^b^
40	7.8 ± 0.02^c^	3.1 ± 0.00^c^	39.74^c^
25	8.3 ± 0.09^d^	2.5 ± 0.02^d^	30.12^d^
pH			
7.0	12 ± 0.26^a^	5.2 ± 0.03^a^	55.00^a^
7.5	11 ± 0.60^b^	5.1 ± 0.12^a^	51.00^a^
6.5	10 ± 0.05^c^	2.9 ± 0.02^b^	29.00^b^
8.0	9.5 ± 0.4^c^	2.6 ± 0.00^c^	27.36^c^
Carbon source			
Glucose	10.0 ± 0.02^a^	6.1 ± 0.07^a^	61.00^a^
Sugarcane bagasse	9.0 ± 0.34^b^	5.0 ± 0.08^b^	55.55^b^
Fructose	9.6 ± 0.06^a^	5.2 ± 0.11^b^	54.16^b^
Corn cob	9.3 ± 0.09 ^b^	4.8 ± 0.34^b^	51.61^c^
Sucrose	8.6 ± 0.28 ^c^	4.2 ± 0.13^c^	48.83^c^
Teff straw	8.3 ± 0.21^c^	3.2 ± 0.26^d^	38.55^d^
Banana peel	7.8 ± 0.00^d^	2.1 ± 0.03^e^	26.92^e^
Nitrogen source			
Peptone	8.2 ± 0.21^a^	5.2 ± 0.33^a^	63.41^a^
Ammonium nitrate	8.0 ± 0.41^a^	4.1 ± 0.23^c^	51.25^b^
Yeast extract	9.9 ± 0.03^b^	4.7 ± 0.02^b^	47.47^b^
Sodium nitrate	9.6 ± 0.09^b^	4.3 ± 0.12^c^	44.79^c^
Casein	8.7 ± 0.09^c^	3.5 ± 0.03^d^	40.22^d^
Ammonium sulphate	7.6 ± 0.11^d^	3.0 ± 0.53^e^	39.47^d^

Growth parameters were maintained at pH 7, 370 °C, 48 h and 150 rpm except for pH and Temperature tests

* Biomass weight takes account of PHB weight

Results are means of 2 trials done in triplicates ± standard deviation

Different superscript letters indicate significant difference within the same column at P  <  0.05

Among the organic and inorganic nitrogen-sources tested for the production of PHB by isolate AWW, peptone (63%) was the best nitrogen source. Ammonium nitrate led to 51% PHB production which was equivalent to yeast extract (48%). PHB production was least with casein (40%) and ammonium sulfate (40%) (Table 3).

**Fig. 3:**
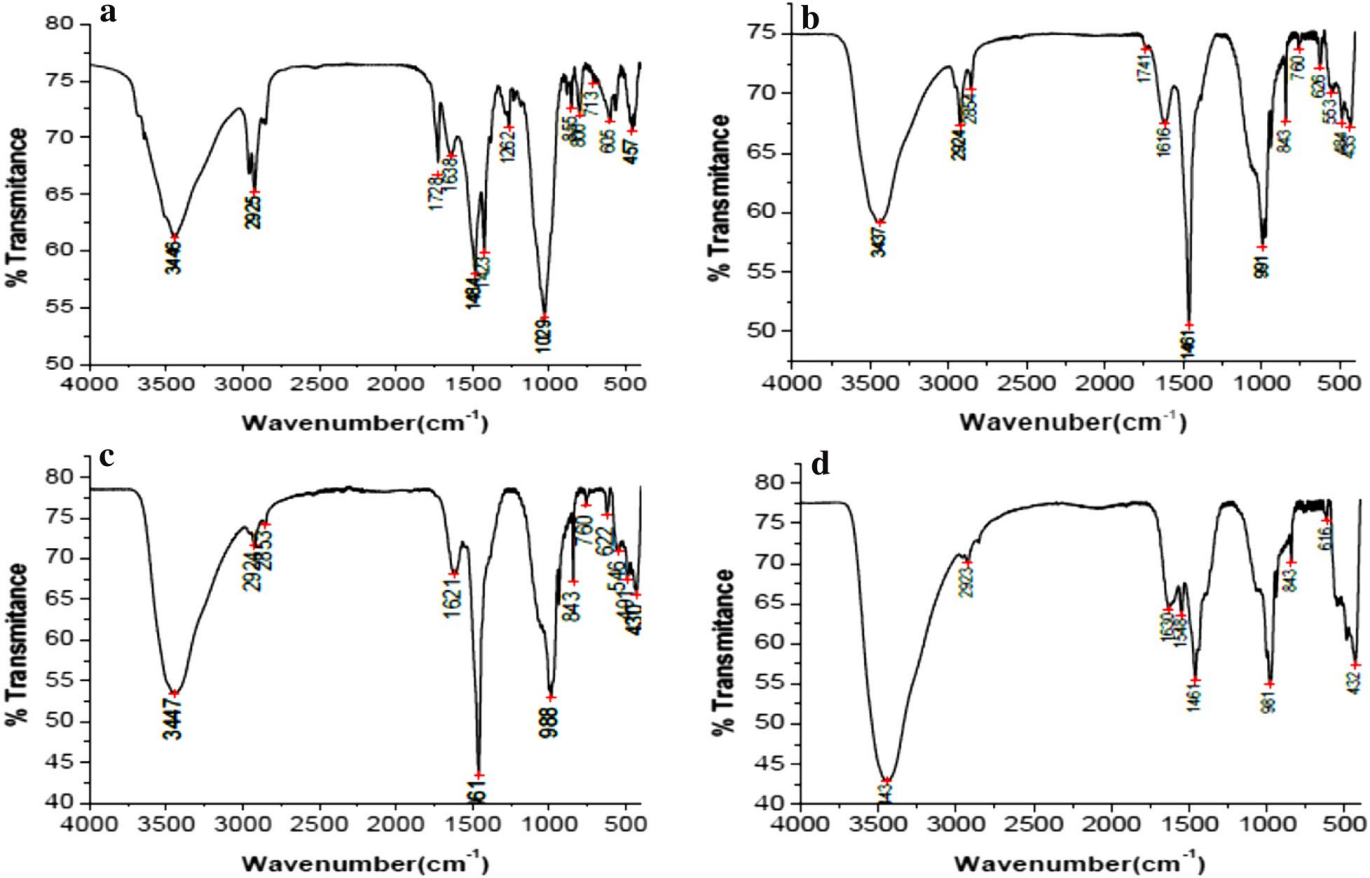
FTIR analysis of polyhydroxybutyrate polymer extracted from isolate AWW grown in medium containing sugarcane bagasse (**a**), corn cob (**b**), teff straw (**c**) and banana peel (**d**) as carbon sources

**Fig. 4:**
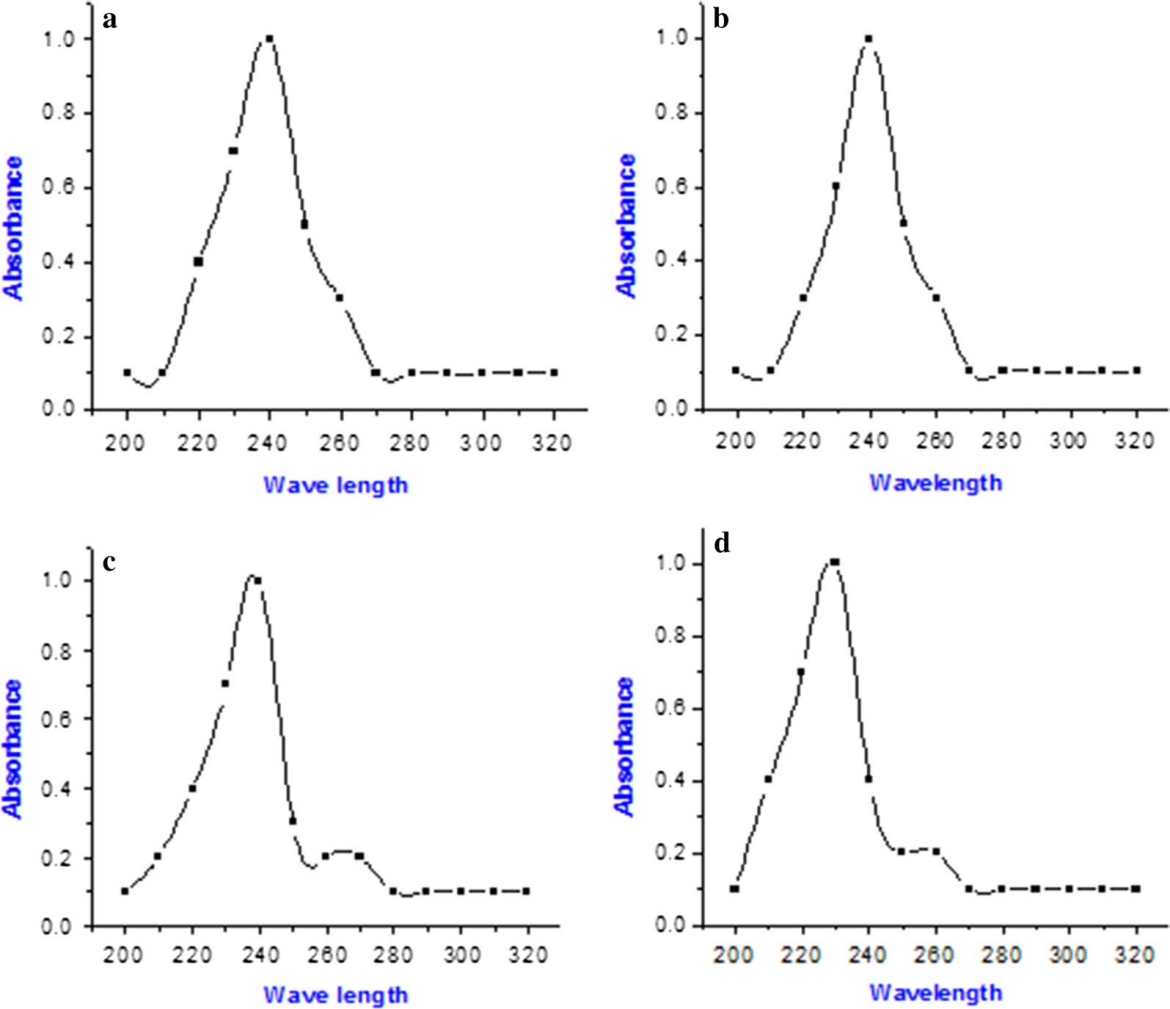
UV–Vis spectrophotometer scanning spectrum of PHB compounds extracted from isolate AWW grown in sugarcane bagasse (**a**), corn cob (**b**) teff straw (**c**) and banana peel (**d**)

The extracted PHB samples were evaluated for identification of their functional groups through FTIR analysis (Fig. 3). The functional groups were identified as –OH, –CH_2_, C=O ester, C=O amide protein, N–H amide protein, CH_3_, –C–O– and alkyl halides. UV–Vis spectrophotometer scanning revealed that, the absorbance peaks were 240 for sugarcane bagasse, corn cob and teff straw and 230 for banana peel (Fig. 4).

**Fig. 5:**
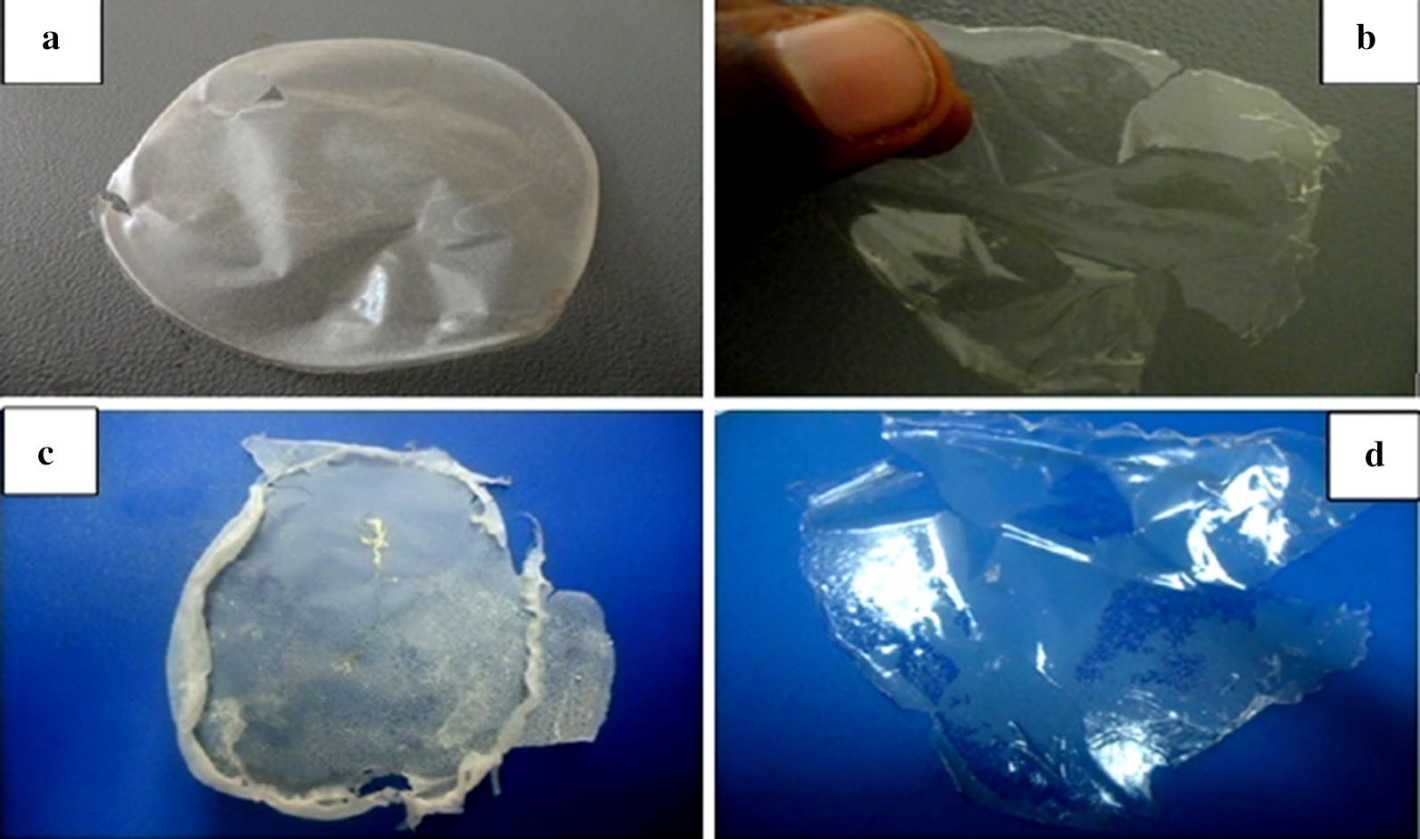
Appearance of biodegradable plastic produced from sugarcane bagasse (**a**), corn cob (**b**) teff straw (**c**) and banana peel (**d**) by isolate AWW

**Fig. 6:**
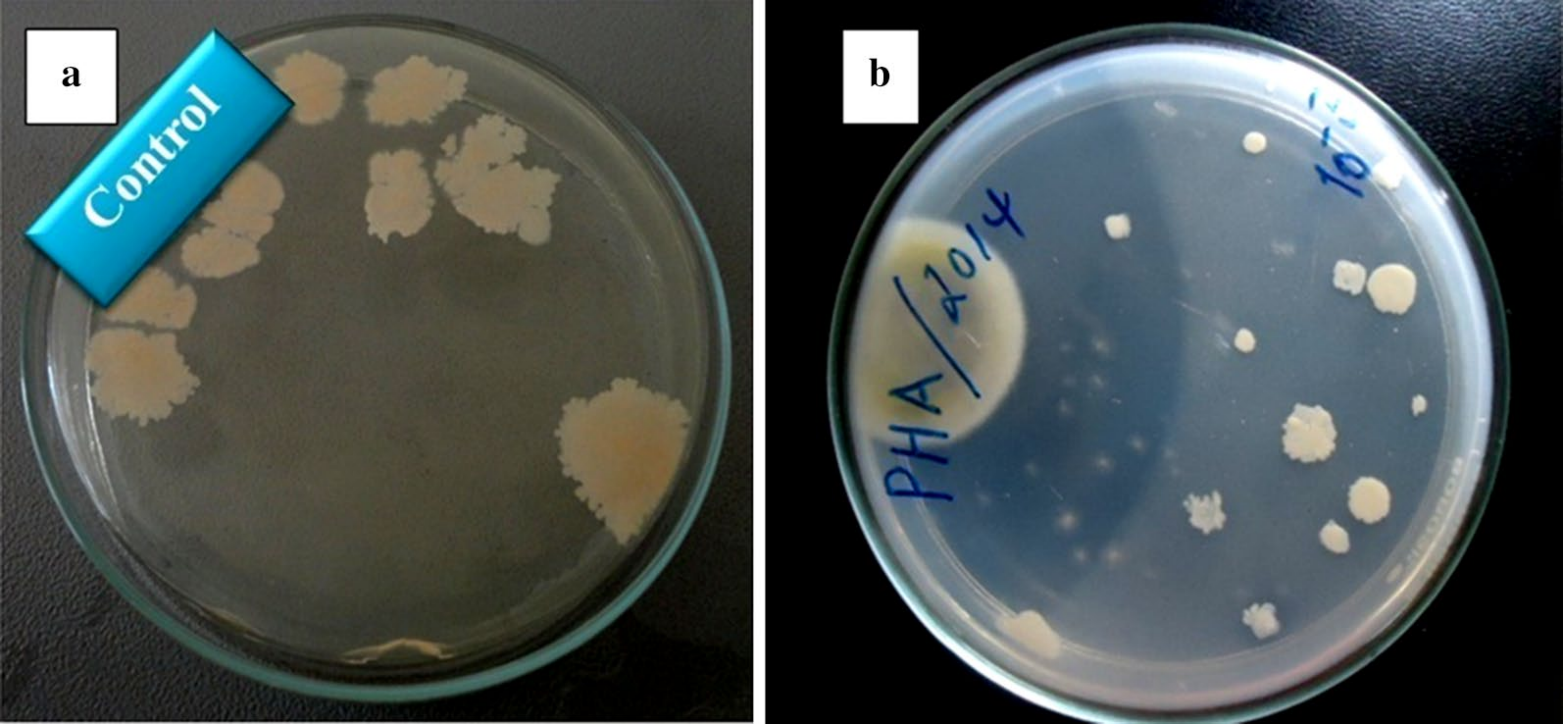
Degradation study of PHB by clear zone method in mineral salt medium devoid of PHB polymer (**a**) and mineral salt medium containing PHB polymer (**b**)

The biodegradable plastic prepared from PHB extract produced from sugarcane bagasse and the clear zone formed by the PHB degradation with soil born bacteria are shown in Figs. 5 and 6 respectively.

## Discussion

In the present study potential PHB accumulating bacteria were isolated from diverse sources and potential strains were selected for further studies (Table 1). Most of the potential isolates were Bacilli. *Bacillus* sp are reported to be ideal PHB producers in many previous studies [17, 18].

The optimum growth and the maximum PHB accumulation by isolate AWW happened at 48 h. (Table 2 and Fig. 2). This shows biomass and PHB production were concomitant with growth conditions and PHB production of a particular strain is related to its biomass. As biomass increases the bacteria starts accumulating PHB to the maximum level and the accumulated PHB decreases after the peak biomass production. This might be due to nutrient depletion, which forces the bacteria to use the accumulated PHB as energy source [19].

The highest yield of PHB was obtained with glucose (61%) after 48 h incubation (Table 3). Comparable result were reported by Adwitiya et al. [20] from *R. sphberoides* N20 and Gomez et al. [21] from *Alcaligenes latus* using glucose as carbon source. Glucose is an easily assimilable carbon source that encourages bacteria to produce more PHB [22–24].

Pre-treated sugarcane bagasse (56%) was the best cheap carbon source followed by corn cob (52% PHB) (Table 3). Similar results were reported by Yu et al. [6], who obtained 54% PHB using bagasse hydrolytes from *Cupriavidus necator*. Paramjeet et al. [25] obtained 60% PHB from sugarcane bagasse by *Pseudomonas aeruginosa.* On the other hand teff straw and banana peel resulted in less biomass and PHB production (Table 3). A striking observation was made in banana peel in which the proportion of 27% PHB was far less than other carbon sources. It seems that, banana peel could support the growth of the bacterium but does not contribute much for PHB production. This might be due to lack of excess carbon source in the medium reflected by the relatively less reducing sugar content of pretreated banana peel.

The optimum temperature for growth and accumulation of PHB by isolate AWW was 37 °C (Table 3). The PHB and biomass yields increased till 37 °C and sharply declined at temperature extremes (Table 3). Noha et al. [24] also reported maximum cell density and PHB accumulation at 37 °C after 48 h. The alteration in the PHB content by temperature variance can be due to the fact that extreme temperatures slow down the metabolic activity (enzyme activity) of microorganisms that ultimately reduces their ability to produce PHB.

The maximum PHB production by isolate AWW occurred at pH range of 7.0–7.5 (Table 3) with 55 and 51% PHB production (Table 3). This result is in line with the reports by Hong et al. [8] in which optimum microbial growth and PHB production occurred in the pH range of 6.0–7.5.

The maximum PHB production percentage per dry cell weight was achieved with peptone as a nitrogen source followed by ammonium nitrate with PHB contents of 63 and 51% respectively (Table 3). This might be due to the relatively low nitrogen content of peptone that resulted in increased C/N ratio which in turn favored higher PHB accumulation [10]. The least cell dry weight and PHB content were achieved with ammonium sulphate. It has been reported by Paramjeet et al. [25] that, the nitrogen concentration in bacteriological media highly influences the production of intracellular PHB.

In the FTIR analysis results, the peaks at 3446, 3406, 3443 and 3443 cm^−1^ indicated stretching strong H bond created by the terminal OH groups found in sugarcane bagasse, corn cob, teff straw and banana peel produced PHB samples respectively (Fig. 3). Similar results have been reported in other works [26, 27]. The peaks at 2924/2925 and 2923/2924 cm^−1^ are assigned to C–H stretching methyl and methylene groups respectively. These are comparable with results obtained by Kumalaningsih et al. [28] (2925.81 cm^−1^) and Anish et al. [29] (2932 cm^−1^).

According to Randriamahefa et al. [30] the absorption bands of 1728 and 1741 cm^−1^ (observed in Fig. 3) are PHA marker bands allocated to carbonyl C= O stretches of the ester groups located in the chain of exceedingly ordered crystalline structures. These peaks were comparable with the standard peaks 1728 and 1740 cm^−1^ to scl-PHA and mcl-PHA respectively [8]. A short chain length PHA (scl-PHA) represents 3–5 carbon atoms and medium-chain length PHA (mcl-PHA) 6–14 carbon atoms monomer units [2]. The peaks at 1638, 1621, 1630 and 1616 cm^−1^ indicate a weak C=O bond extended for conjugated carbonyl or amide group for the sample sugarcane bagasse, corn cob, teff straw and banana peel respectively. This was similar with peaks obtained by Kumalaningsih et al. [28].

The peak at 1548 cm^−1^ for sample BP indicates the presence of N–H amide protein in the polymer. This was similar with the result reported 1560.3 cm^−1^ by Kumalaningsih et al. [28]. While series peak at 1484, 1461, 1461 and 1461 cm^−1^ for the samples sugarcane bagasse, corn cob, teff straw and banana peel respectively accounts for –CH_2_. The peaks 1262, 1029 cm^−1^ represents –C–O– polymeric group in the sample sugarcane bagasse. Stretching other peaks (991,981, 855, 843, 800, 760, 626, 622, 616, 605, 553, 546, 491, 484, 457, 434 and 430 cm^−1^) correspond to the presence of Alkyl halides [14]. These all prominent absorption bands confirm that the polymer extracted from all samples were poly-β-hydroxybutyrate.

UV–Vis scanning of the extracted polymers showed peaks between 230 and 240 nm readings (Fig. 4). This peak range indicates the occurrence of PHB [14].

The plastic nature and biodegradability of the extracted polymer was confirmed by preparing sample plastic film (Fig. 5) and the clear zone formed by soil born bacteria (Fig. 6). PHBs are degraded by the action of microbial enzyme into water-soluble forms [31, 32]. Mergaert et al. [33] and his co-worker described 295 strains of PHB and P (3HB-co-3HV) copolymer degrading soil microorganisms.

## Conclusion

The results of this study confirmed that cheaply available agro-residues can be used for the production of PHB serving triple purposes of reducing the cost of biodegradable plastics, reducing environmental pollution problems caused by conventional plastics and solving disposal problem of the agricultural wastes.

## Abbreviations

ASS: AMU sewage sludge
AMU: Arba Minch University
AWW: Arba minch waste water
FPS: fish processing soil
KAS: Kulfo agriculture soil
KFS: Kulfo field soil
KIS: Kulfo irrigation soil
LAW: Lake Abaya water
LCW: Lake Chamo water
mcl-PHA: Medium-chain length PHA
PHBs: polyhydroxybutyrates
scl-PHA: short chain length PHA
SFS: Sile field soil
SRS: Sile river sludge
